# Guest Editorial: Breast Milk: An Optimal Food

**DOI:** 10.1289/ehp.112-a722

**Published:** 2004-09

**Authors:** Jenny Pronczuk, Gerald Moy, Constanza Vallenas

**Affiliations:** World Health Organization Geneva, Switzerland E-mail: pronczukj@who.int

Human breast milk offers the optimal nutrition for all infants and provides immunological, developmental, psychological, economic, and practical advantages when compared to artificial feeding. For proper growth, development, and health, infants should be exclusively breast-fed with no other food or drink—not even water—for their first 6 months of life [World Health Organization (WHO) 2001]; they should then receive nutritionally adequate and safe complementary foods while breast-feeding continues up to 24 months of age or beyond.

Given the considerable benefits of breast-feeding for mothers and children everywhere, special efforts are being undertaken by the WHO and partners to promote it in all countries. The *Global Strategy for Infant and Young Child Feeding* (WHO 2003) recommends critical interventions such as the implementation and monitoring of the International Code of Marketing of Breast-milk Substitutes and the subsequent relevant World Health Assembly resolutions; the adoption and monitoring of maternity entitlements consistent with the International Labour Organization (ILO) Maternity Protection Convention (ILO 2000); and the expanded implementation of the WHO/UNICEF Baby-Friendly Hospital Initiative (WHO/UNICEF 1992). Education of women as well as men about the benefits of breast-feeding is being promoted to establish broader social acceptance of and support for breast-feeding.

New knowledge is emerging on the importance of breast-feeding and the origin of some adult diseases. Breast-feeding may be related to the prevention of diabetes, heart disease, and other diseases that appear in adulthood.

When there is a risk of infectious and toxic agents being present in human milk, however, specific recommendations may apply. To address these and other concerns, the WHO promotes collaborative research studies and develops guidance on the prevention of exposure and the reduction of risk. This represents a challenging task because guidelines must address global public health issues while taking into account the needs of countries and peoples with different health care, sociocultural, and economic conditions.

Among the infectious agents, tuberculosis, hepatitis B virus (HBV), and human immunodeficiency virus (HIV) are considered the main global threats to the health of mothers and infants. In the case of maternal tuberculosis, infants should remain with their mothers and be immunized with BCG (bacillus Calmette-Guérin) as soon as possible after birth to protect them from meningeal and pulmonary tuberculosis. Mothers are treated with the standard short course antibiotic regimes compatible with breast-feeding (WHO 1998a). HBV is another major public health problem. Breast-feeding seems to be an additional mechanism by which infants acquire HBV infection; however, the risk associated with breast-feeding is negligible compared with that of exposure to maternal blood and body fluids at birth. In industrialized countries pregnant mothers are screened for hepatitis B surface antigen, and infants are treated with specific hyperimmune globulin and HBV vaccine, but in developing countries only the routine immunization of infants with HBV vaccine is possible and breast-feeding is still recommended (WHO 1996a). Mother-to-child transmission of HIV is the most significant source of HIV infection in children, and 5–20% of infants born to HIV-infected mothers may acquire it through breast-feeding. Given the need to reduce the risk of transmission to infants while minimizing the risk of other causes of morbidity and mortality, current guidelines state that when replacement feeding is acceptable, feasible, affordable, sustainable, and safe, HIV-infected mothers should avoid breast-feeding completely (WHO/UNICEF/UNFPA/UNAIDS 2003). When these conditions are not present, HIV-infected women who choose to breast-feed are recommended to do so exclusively for the first few months. Then, over a period of a few days to a few weeks, they may gradually stop breast-feeding (exclusive breast-feeding with early cessation), provided the conditions for replacement feeding or other breast-milk options are in place.

There is a myriad of potential chemical contaminants that can be detected in breast milk as analytical methods become ever more sensitive. Most research studies deal with dioxins, polychlorinated biphenyls (PCBs), and organochlorine pesticides. These chemicals belong to the group of persistent organic pollutants (POPs) and are being studied in view of their potential endocrine-disrupting effects. Studies undertaken by the WHO over the past 15 years on dioxins and PCBs demonstrated that in most countries levels of these chemicals in breast milk continue to decrease (WHO 1988, 1989, 1996b). The latest study (Van Leeuwen and Malisch 2002) concluded that in view of this trend, breast-feeding should be encouraged and promoted because of its multiple benefits for the overall health and development of infants. A safety evalutation by the WHO (2002) noted that for PCBs, the exposure of infants through breast milk may be less important than exposure *in utero* and that most of the subtle effects observed are associated more with transplacental exposure than with exposure through breast-feeding.

The risk assessment of selected organochlorine contaminants in breast milk undertaken by the WHO in 1998 showed that DDT concentrations were higher in developing countries and that hexa-chlorobenzene levels were higher in industrialized countries (WHO 1998b). However, it was stressed that the primary preventive measures to control and reduce the introduction of organochlorine compounds in the environment were the most effective means to eliminate and minimize contaminants in breast milk. Under the Stockholm Convention (United Nations Environment Programme 2001), which was ratified in May 2004, the production and emission of the first group of 12 POPs are to be reduced or eliminated.

Tobacco smoking deserves special consideration because it increases the exposure of mothers and infants to a large number of toxicants, including pesticide residues and known carcinogens, and is linked to reduced duration of breast-feeding and higher levels of abdominal distress in the child. Women who smoke are encouraged to breast-feed and to eliminate cigarette use during pregnancy and lactation.

In view of existing and new information available on infectious and chemical breast milk contaminants, appropriate mechanisms for assessing, preventing, and communicating potential health risks should be considered. Risk communication is of paramount importance—“do not hide, do not scare”—and should enable the informed choice of the mother. In most cases, mothers can and should be reassured that breast milk is by far the best food to give to their babies.

## Figures and Tables

**Figure:**
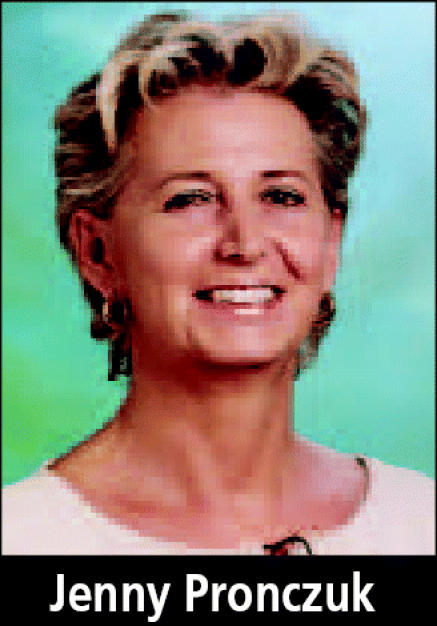


**Figure:**
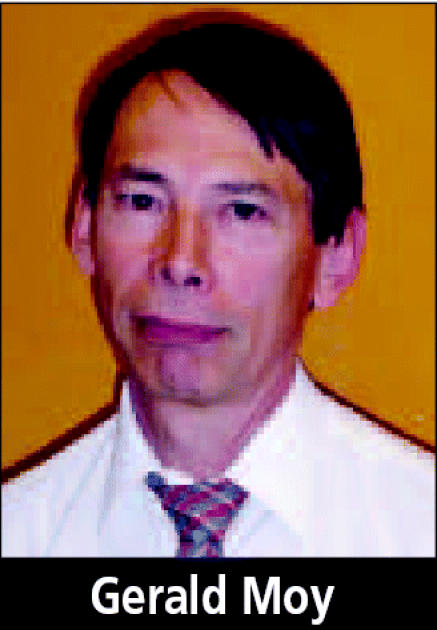


**Figure:**
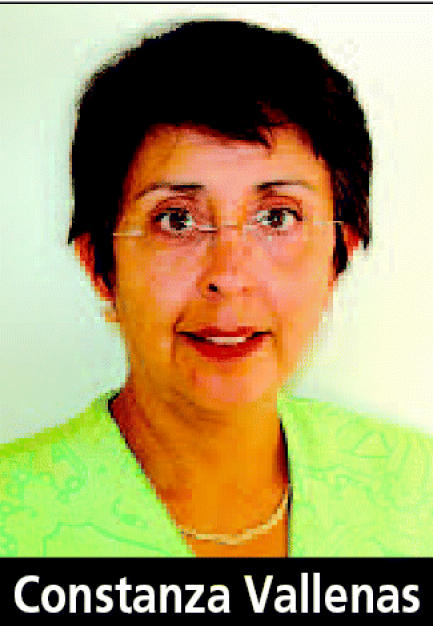


## References

[b1-ehp0112-a00722] ILO (International Labour Organization) 2000. C183 Maternity Protection Convention, 2000. Available: http://www.ilo.org/ilolex/cgi-lex/convde.pl?C183 [accessed 10 August 2004].

[b2-ehp0112-a00722] United Nations Environment Programme 2001. Stockholm Convention on Persistent Organic Pollutants (POPS). Available: http://www.pops.int [accessed 11 August 2004].

[b3-ehp0112-a00722] Van Leeuwen FXR, Malisch R (2002). Results of the third round of WHO-coordinated exposure study on the levels of PCBs, PCDDs and PCDFs in human milk. Organohalogen Compounds.

[b4-ehp0112-a00722] WHO 1988. Assessment of Health Risks in Infants Associated with Exposure to PCBs, PCDDs and PCDFs in Breast Milk: Report on a WHO Working Group, Abano Terme/Padua, 16–19 February 1987 (Grandjean P, ed). Environmental Health Series 29. Copenhagen:World Health Organization Regional Office for Europe.

[b5-ehp0112-a00722] WHO 1989. Levels of PCBs, PCDDs and PCDFs in Breast Milk: Results of WHO-Coordinated Interlaboratory Quality Control Studies and Analytical Field Studies (Yrjänheikki E, ed). Environmental Health Series 34. Copenhagen:World Health Organization Regional Office for Europe.

[b6-ehp0112-a00722] WHO 1996a. Hepatitis B and Breastfeeding. Child and Adolescent Health and Development Update No 22. Available: http://www.who.int/child-adolescent-health/publications/NUTRITION/Up_22.htm [accessed 10 August 2004].

[b7-ehp0112-a00722] WHO 1996b. Levels of PCBs, PCDDs and PCDFs in Human Milk: Second Round of WHO-Coordinated Exposure Study. Environmental Health in Europe No 3. Bilthoven, Netherlands:World Health Organization European Centre for Environment and Health.

[b8-ehp0112-a00722] WHO 1998a. Breastfeeding and Maternal Tuberculosis. Child and Adolescent Health and Development Update No 23. Available: http://www.who.int/child-adolescent-health/publications/NUTRITION/Up_23.htm [accessed 10 August 2004].

[b9-ehp0112-a00722] WHO 1998b. GEMS/Food International Dietary Survey: Infant Exposure to Certain Organochlorine Contaminants from Breast Milk—A Risk Assessment (Schutz D, ed). WHO/FSF/FOS/98.4. Geneva:Food Safety Unit, Programme of Food Safety and Food Aid, World Health Organization.

[b10-ehp0112-a00722] WHO 2001. The Optimal Duration of Exclusive Breastfeeding. Report of an Expert Consultation. WHO/NHD/01.09 and WHO/FCH/CAH/01.24. Geneva:World Health Organization.

[b11-ehp0112-a00722] WHO 2002. Safety Evaluation of Certain Food Additives and Contaminants. WHO Food Additives Series 48. Geneva:World Health Organization, 311–316.

[b12-ehp0112-a00722] WHO 2003. Global Strategy for Infant and Young Child Feeding. Geneva:World Health Organization.

[b13-ehp0112-a00722] WHO/UNICEF 1992. The Global Criteria for the WHO/UNICEF Baby-Friendly Hospital Initiative. In: Baby-Friendly Hospital Initiative. Part II. Hospital Level Implementation. Geneva:World Health Organization.

[b14-ehp0112-a00722] WHO/UNICEF/UNFPA/UNAIDS 2003. HIV and Infant Feeding: Guidelines for Decision-makers. Geneva:World Health Organization. Available: http://www.who.int/child-adolescent-health/publications/NUTRITION/ISBN_92_4_159122_6.htm [accessed 10 August 2004].

